# MND Phenotypes Differentiation: The Role of Multimodal Characterization at the Time of Diagnosis

**DOI:** 10.3390/life12101506

**Published:** 2022-09-27

**Authors:** Giuseppe Meo, Pilar M. Ferraro, Marta Cillerai, Chiara Gemelli, Corrado Cabona, Federico Zaottini, Luca Roccatagliata, Flavio Villani, Angelo Schenone, Claudia Caponnetto

**Affiliations:** 1 Department of Neurology, IRCCS Ospedale Policlinico San Martino, 16132 Genoa, Italy; 2 Department of Neuroscience, Rehabilitation, Ophthalmology, Genetics, Maternal and Child Health (DINOGMI), University of Genoa, 16126 Genoa, Italy; 3 Division of Clinical Neurophysiology and Epilepsy Center, IRCCS Ospedale Policlinico San Martino, 16132 Genoa, Italy; 4 Department of Radiology, IRCCS Ospedale Policlinico San Martino, 16132 Genoa, Italy; 5 Department of Neuroradiology, IRCCS Ospedale Policlinico San Martino, 16132 Genoa, Italy; 6 Department of Health Sciences (DISSAL), University of Genoa, 16126 Genoa, Italy

**Keywords:** motor neuron diseases, differential diagnosis, multimodal characterization, amyotrophic lateral sclerosis

## Abstract

**Simple Summary:**

Diverse motor neuron diseases (MNDs) are associated with distinct survival rates, so their early differentiation is pivotal to gain a more reliable prognosis estimation in clinical and research settings. In this study, we therefore evaluated whether a multimodal characterization approach embedding clinical, cognitive/behavioral, genetic, and neurophysiological data may improve the discrimination of pure/predominant upper motor neuron (pUMN) and pure/predominant lower motor neuron (pLMN) disease forms from classic amyotrophic lateral sclerosis (ALS) already by the time of diagnosis. Our results suggest common cognitive and genetic features across the distinct MND phenotypes, but also demonstrate that highly specific clinical and neurophysiological measures provide valuable tools for an early discrimination between more benign and more aggressive disease forms.

**Abstract:**

Pure/predominant upper motor neuron (pUMN) and lower motor neuron (pLMN) diseases have significantly better prognosis compared to amyotrophic lateral sclerosis (ALS), but their early differentiation is often challenging. We therefore tested whether a multimodal characterization approach embedding clinical, cognitive/behavioral, genetic, and neurophysiological data may improve the differentiation of pUMN and pLMN from ALS already by the time of diagnosis. Dunn’s and chi-squared tests were used to compare data from 41 ALS, 34 pLMN, and 19 pUMN cases with diagnoses confirmed throughout a 2-year observation period. Area under the curve (AUC) analyses were implemented to identify the finest tools for phenotypes discrimination. Relative to ALS, pLMN showed greater lower limbs weakness, lower UMN burden, and progression rate (*p* < 0.001–0.04). PUMN showed a greater frequency of lower limbs onset, higher UMN burden, lower ALSFRS-r and MRC progression rates (*p* < 0.001–0.03), and greater ulnar compound muscle action potential (CMAP) amplitude and tibial central motor conduction time (CMCT) (*p* = 0.05–0.03). The UMN progression rate was the finest measure to identify pLMN cases (AUC = 90%), while the MRC progression rate was the finest tool to identify pUMN (AUC = 82%). Detailed clinical and neurophysiological examinations may significantly improve MNDs differentiation, facilitating prognosis estimation and ameliorating stratification strategies for clinical trials enrollment.

## 1. Introduction

Motor neuron diseases (MNDs) are a group of heterogeneous neurodegenerative syndromes characterized by the progressive deterioration of upper (UMN) and/or lower motor neurons (LMN), leading to advancing muscular paralysis and death [1].

It is now widely accepted that the clinical spectrum of MNDs is extremely variable, ranging from classic amyotrophic lateral sclerosis (ALS) to pure/predominant LMN (pLMN) disease forms, such as progressive muscular atrophy (PMA), flail arm and flail leg phenotypes, and pure/predominant UMN syndromes, including primary lateral sclerosis (PLS) and pyramidal phenotypes [2].

Notably, while detailed clinical studies of these diverse syndromes are still rare, it has been recently shown that pUMN and pLMN phenotypes exhibit significantly longer survival compared to classic ALS [3], suggesting that the early identification of these syndromes may have remarkable prognostic relevance.

On the other hand, accurate MNDs differentiation at the time of diagnosis might be particularly challenging for multiple reasons: the initial clinical manifestations are often subtle and heterogenous [4], and additionally, by definition, confirmed diagnoses of more benign syndromes require long observation periods; flail arm and flail leg syndromes, for example, are characterized by functional involvement confined to the onset limbs for at least 12 months [2,3], and PLS and PMA are defined by the absence, respectively, of LMN and UMN degeneration for up to 4 years from symptom onset [5,6].

In light of all the aforementioned observations, it becomes clear why a considerable diagnostic delay is frequently observed in benign MND phenotypes, with negative effects on reliable prognosis estimation, patient management, and clinical trials stratification strategies.

In this context, the aim of our study was to retrospectively analyze a large sample of MND patients with ALS, pLMN, and pUMN diagnoses confirmed throughout a 2-year observation period in order to: (a) better depict the diverse MND phenotypes using a multimodal characterization approach embedding clinical, cognitive/behavioral, genetic, and neurophysiological features, and (b) test whether the information derived from such an approach would have been able to improve the discrimination of pLMN and pUMN cases from classic ALS patients already at the time of diagnosis.

## 2. Materials and Methods

### 2.1. Participants

As part of a larger study established in 2019 and still in operation, all patients diagnosed with MND at our center, who agree to participate, undergo a thorough battery of baseline evaluations including: neurological history, neurophysiological exams, clinical assessments, extra-motor symptoms examinations, and genetic screening.

Neurophysiological data are obtained using Synergy Software. Compound muscle action potentials (CMAPs) are acquired from the upper limbs’ median and ulnar nerves and from the lower limbs’ peroneal and tibialis posterior nerves, both proximally and distally. Motor-evoked potentials (MEPs) are elicited through a magnetic stimulator (Magstim) and, from the bilateral abductor pollicis brevis and tibialis anterior muscles, the central motor conduction time (CMCT) is calculated.

Experienced neurologists perform all the clinical assessments. Onset site (bulbar vs. spinal), limbs (upper vs. lower), side (right vs. left vs. bilateral), and muscles involvement (proximal vs. distal), as well as disease duration, are recorded.

Disease severity is assessed using the ALS Functional Rating Scale-revised (ALSFRS-r) [7], muscular weakness is evaluated using the Medical Research Council (MRC) scale [8], and the severity of UMN involvement is graded using the UMN score [9]. For each scale an additional baseline rate of progression is calculated as follows: (maximum score of the scale—actual patient score at the time of examination)/months from symptom onset to examination.

Experienced neuropsychologists perform all the extra-motor symptoms examinations. Cognitive and behavioral alterations are evaluated using the Italian version of the Edinburgh Cognitive and Behavioral ALS Screen (ECAS) [10]. Anxiety and depressive symptoms are assessed using the Hospital Anxiety and Depression Scale [11].

Genetic screening includes both C9orf72 GGGGCC repeat length analysis and next-generation sequencing (NGS) analysis with two multiple gene panels including, respectively, 23 and 27 genes known to be associated or possibly associated with ALS. The genes included in the panels are given in Supplementary Table S1.

Patients are then followed longitudinally with clinical and behavioral/mood examinations approximately every 3 months, and with cognitive evaluations approximately every 6 months.

From this large dataset, the present retrospective study selected all the MND cases fulfilling, both at first examination and during a 2-year observation period, current criteria for the following phenotypes: classic ALS, pLMN (flail arm, flail leg, and pure LMN), and pUMN (pyramidal and pure UMN) [3].

### 2.2. Statistical Analyses

All data were analyzed using CRAN R Version 3.4.1 (https://cran.r-project.org/, accessed on 16 May 2022), and the statistical significance threshold was set to *p* ≤ 0.05.

Continuous variables were compared between ALS, pLMN, and pUMN groups using the Kruskal–Wallis test and nonparametric pairwise multiple comparisons between ALS patients and pLMN patients, as well as between ALS patients and pUMN patients’ were subsequently run using the Dunn’s test with Bonferroni adjustment to control for the familywise error rate. Categorical variables were compared between patient groups using the chi-squared test.

Afterwards, receiver operating characteristics (ROC) curve and area under the curve (AUC) analyses were implemented to evaluate the sensitivity and specificity of differentiating features in the discrimination between pLMN and pUMN phenotypes and classic ALS.

According to current references [12], AUC values lower than 0.7 suggested no discrimination, AUC values from 0.7 to 0.8 were considered acceptable, AUC values from 0.8 to 0.9 were considered excellent, and AUC values greater than 0.9 were considered outstanding.

Finally, in order to explore whether features discriminating pLMN and pUMN patients from classic ALS would have been further associated with a more stable clinical course, linear regression models were applied to test the role of those measures in predicting the degree of functional impairment at the end of the observation period (evaluated using the ALFRS-r score).

## 3. Results

### 3.1. Demographic, Onset, Clinical, Neurophysiological, Extra-Motor, and Genetic Data Comparisons

Demographic, onset, clinical, neurophysiological, extra-motor, and genetic features of ALS, pLMN, and pUMN patients are summarized in Table 1.

No significant differences were observed between pLMN and ALS cases in terms of demographic, onset, and neurophysiological characteristics.

As regards clinical features, as expected, pLMN cases showed a lower frequency of clinically probable (*p* < 0.001) and clinically probable–laboratory supported (*p* = 0.007) El Escorial diagnoses, and a greater frequency of *suspected* El Escorial categories (*p* < 0.001) compared to ALS patients. Additionally, pLMN cases exhibited greater right lower limb muscular weakness (lower MRC scores, *p* = 0.04) and inferior total and regional UMN burden (lower total UMN score *p* < 0.001, upper limbs UMN score *p* < 0.001, and lower limbs UMN score *p* < 0.001), as well as lower UMN rate of progression (*p* < 0.001) (Table 1).

No significant differences were observed between pUMN and ALS cases in terms of demographic variables. As regards onset characteristics, pUMN patients exhibited a greater frequency of symptom onset in the lower limbs (*p* = 0.02) (Table 1).

Concerning clinical features, by definition, pUMN cases showed a greater frequency of possible El Escorial diagnoses (*p* < 0.001). Furthermore, they manifested less severe overall functional and muscular weakness worsening (lower ALSFRS-r rate of progression *p* = 0.01, lower MRC rate of progression *p* < 0.001) as well as greater total and regional UMN burden (higher total UMN score *p* = 0.03, upper limbs UMN score *p* = 0.04, lower limbs UMN score *p* = 0.01, cranial UMN score *p* = 0.03). In terms of neurophysiological features, pUMN cases exhibited higher right ulnar nerve CMAP amplitude (*p* = 0.05) as well as increased right tibialis anterior CMCT (*p* = 0.03).

Information regarding family history was available in all patients, while genetic screening was performed, respectively, in 46.34% of ALS, 58.82% of pLMN, and 42.10% of pUMN cases. No differences were observed between, respectively, pLMN and ALS cases, as well as between pUMN and ALS cases, in the frequency of either positive family histories for ALS and/or other neurodegenerative diseases and known genetic mutations.

Extra-motor symptom evaluations were available in 85.36% of ALS, 85.29% of pLMN, and 73.68% of pUMN cases. No significant differences were observed between pLMN and ALS cases in terms of extra-motor features, while pUMN cases exhibited more severe depressive symptoms (higher total HADS score *p* = 0.01 and depression HADS score *p* = 0.005) compared to ALS patients.

### 3.2. ROC Analysis

**Figure 1:**
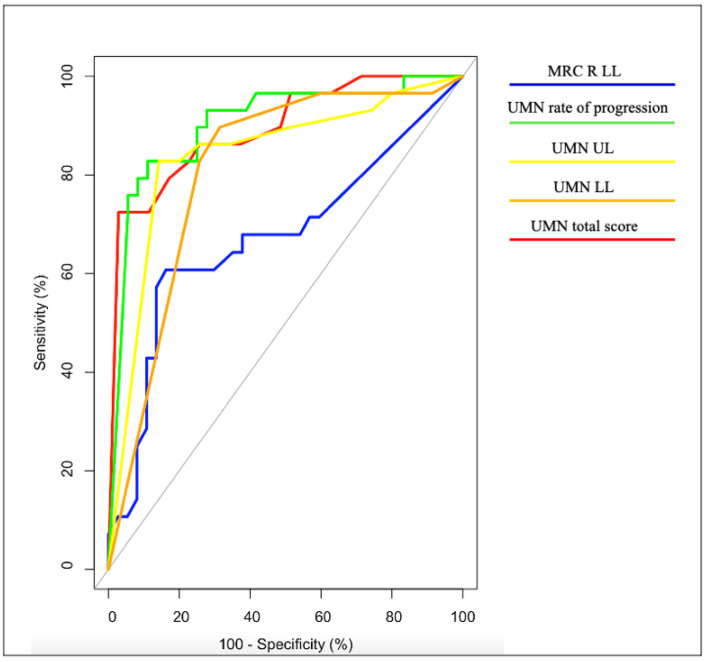
ROC curves: differentiation between ALS and pLMN patients.

The ROC analysis demonstrated the UMN rate of progression to be the finest tool to differentiate pLMN from ALS patients, with AUC = 90%, sensitivity = 83%, and specificity = 89% (Figure 1, Table 2).

Total, upper limbs and lower limbs UMN scores exhibited excellent AUC values ranging from 81% to 89%, with sensitivity values ranging from 72% to 90% and specificity values ranging from 69% to 97%, while right lower limb weakness showed no acceptable discriminatory values, with AUC = 67%, sensitivity = 61%, and specificity = 84%.

**Figure 2:**
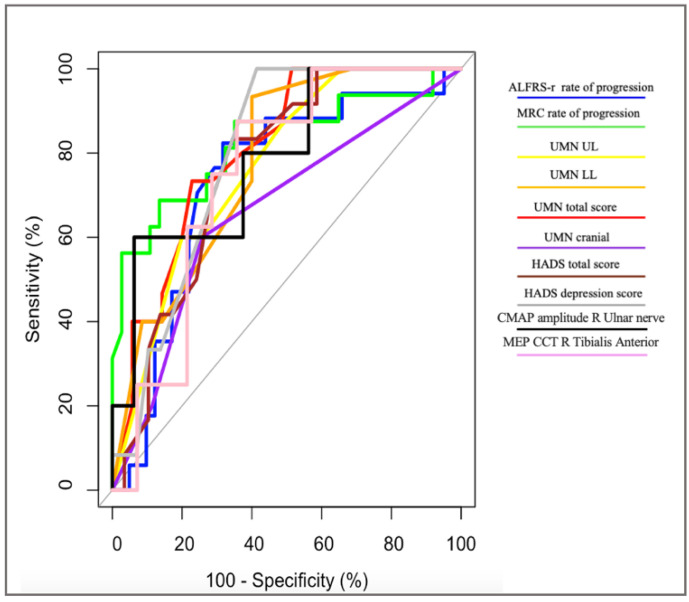
ROC curves: differentiation between ALS and pUMN patients.

Similarly, the ROC analysis revealed the MRC rate of progression to be the most accurate tool for the discrimination between pUMN and ALS patients, with AUC = 82%, sensitivity = 69%, and specificity = 86% (Figure 2, Table 3).

Total UMN and HADS Depression scores showed excellent AUC values ranging from 80% to 81%, with sensitivity values ranging from 73% to 100% and specificity values ranging from 77% to 59%. Acceptable AUC values, ranging from 74% to 79%, were observed for the ALSFRS-r rate of progression, upper limbs, and lower limbs UMN scores, as well as for the HADS total score, CMAP amplitude of the right ulnar nerve, and CMCT of the right tibialis anterior, with sensitivity values ranging from 60% to 93% and specificity values ranging from 60% to 94%. Cranial UMN score showed no acceptable discriminatory values, with AUC = 66%, sensitivity = 60%, and specificity = 74%.

### 3.3. Regression Analysis with Longitudinal Data

In pLMN patients, less severe right lower limb weakness at the time of diagnosis was a significant predictor of higher ALSFRS-r scores at 2 years (*p* = 0.05), while the other features did not show a significant effect (Table 4).

In pUMN patients, lower ALSFRS-r and MRC rates of progression at the time of diagnosis were significant predictors of higher ALSFRS-r scores at 2 years (*p* = 0.01 and *p* = 0.02, respectively), while the other features did not show a significant effect (Table 4).

## 4. Discussion

To our knowledge, this is the first study embedding detailed clinical and neurophysiological assessments, extra-motor symptoms evaluations, and genetic information to better characterize the whole spectrum of the diverse MND phenotypes as currently classified [2,3] and, more importantly, the first one testing the added value of a multimodal characterization approach for the early discrimination between more benign and more aggressive MND forms.

We observed that, while onset, extra-motor and genetic features were largely overlapping between the diverse phenotypes, and highly specific clinical and neurophysiological measures were able, already at the time of diagnosis, to discriminate pLMN and pUMN from ALS cases with significantly accurate performances. A detailed discussion of the obtained findings is provided below.

### 4.1. Findings in pLMN Patients

Similarly to previous reports [13,14,15], we did not observe significant demographic and disease onset differences between pLMN and ALS patients, a finding which may contribute to explaining why the differential diagnosis between these two conditions can be so challenging at the time of first evaluation.

In accordance with the observation of similar onset features, we further found similar degrees of overall functional impairment (ALSFRS-r scores) in pLMN and ALS cases, a result which is largely in line with previous studies of both flail limb and PMA phenotypes [13,16].

Notably, neurophysiological measures were also largely overlapping across pLMN and ALS patients. While the similar observed CMAP amplitudes suggest common patterns of motor units’ loss and reinnervation during the initial stages of the disease, the absence of significant differences in MEP CMCT measures might be related to the composition of the pLMN group, since not only pure but also predominant LMN disease forms were included.

Conversely, compared to ALS, pLMN cases exhibited greater right lower limb muscular weakness as well as less severe total and regional UMN burden. One factor possibly accounting for the severe lower limbs involvement observed in pLMN cases is the so-called split-leg phenomenon, an uneven atrophy recently found to be more prominent in PMA than in ALS cases [16] and postulated to be largely influenced by peripheral pathophysiological mechanisms. Notably, this measure was further predictive of more severe functional impairment at the end of the study period, suggesting either a potential role of this feature in identifying those pLMN cases more likely to convert to classic ALS phenotypes, or simply a significant impact of lower limb muscular weakness on the course of functional status in pLMN syndromes.

The observation of less severe UMN burden in pLMN patients is in line with their clinical diagnoses; however, while previous studies have reported an absence of significant differences in the frequency of UMN signs across bulbar, cervical, and lumbar regions between flail limb and ALS cases [14], the application of more detailed and quantitative UMN assessments in our study has conversely enabled to highlight significant differences between the two phenotypes.

The ROC analysis further confirmed the relevance of UMN burden differences across groups: the UMN total and regional scores exhibited excellent discriminatory performances, yet the finest tool to differentiate pLMN from ALS cases was the baseline UMN rate of progression, yielding sensitivity values of 83% and specificity values of 89%.

While this finding is largely in line with the general observation that progressive UMN degeneration in pLMN phenotypes is associated with upcoming conversion to ALS, it also provides unprecedented evidence that reliable proxies of such a phenomenon can be gained already at the time of diagnosis, providing early excellent discriminatory tools.

### 4.2. Findings in pUMN Patients

No significant differences were observed between pUMN and ALS cases in terms of demographic variables, a finding largely in line with recent reports showing a lack of differences in both sex ratios [17] and age [18] between these two phenotypes.

Concerning disease onset features, we observed that pUMN patients exhibited a greater frequency of symptom onset in the lower limbs, in accordance with previous studies demonstrating that, compared to ALS, UMN cases are more likely to have a spinal onset disease form [18], with preferential initial lower limbs involvement [19,20].

Accordingly, when neurophysiological measures were compared, pUMN cases further presented increased CMCT of the tibialis anterior relative to ALS, in line with both their defining greater pyramidal involvement and their preferential lower limbs onset site.

Additionally, the observation of higher CMAP amplitudes in pUMN, reaching statistical significance for the ulnar nerve, further confirms the notion that near-normal sized CMAP can be observed in more slowly progressing conditions, in which the effectiveness of reinnervation is less limited [21].

As regards clinical features, pUMN cases showed less severe overall functional and muscular weakness worsening, as revealed by their lower ALSFRS-r and MRC baseline rates of progression. Additionally, when we examined the influence of such measures on longitudinal clinical progression, we observed that they were both predictive of more preserved functional status at the end of the study period, further confirming their potential to differentiate pUMN patients less likely to convert to classic ALS phenotypes.

The lower ALSFRS-r progression rate observed in pUMN cases is in accordance with a previous study from Gordon and colleagues showing milder ALSFRS-R decline across visits in PLS and pyramidal phenotypes compared to ALS cases [18], and further confirm initial reports suggesting that UMN patients exhibit the best prognosis among all the MND classes [3].

The observed lower weakness progression rate, in the absence of noticeable MRC score differences, argues in favor of a time-dependent effect, suggesting that pUMN cases develop weakness symptoms of comparable severity relative to those observed in ALS patients, but over a longer time period. Accordingly, previous studies have shown more preserved muscular strength in UMN cases compared to classic ALS patients during early disease phases [18], while a similar frequency of weakness symptoms has been reported in UMN and ALS patients evaluated later in the disease course [17]. Additionally, the lower muscular strength decline observed in pUMN cases might be further explained when one considers that limb wasting is rarer in UMN patients compared to ALS cases [17]. Finally, it is noteworthy to highlight that similar MRC scores should not hinder different contributing pathological mechanisms, namely, flaccid weakness in ALS and spastic weakness in PLS.

The greater total and regional UMN burden observed in pUMN cases compared to ALS patients is largely in line with their clinical definition. However, as for the MRC examinations, we found a dissociation between baseline scores and progression rates, so that pUMN cases exhibited more severe UMN symptoms but with a rate of progression comparable to that observed in ALS cases, suggesting similar trajectories of increasing UMN burden across these phenotypes.

The ROC analysis further confirmed the relevance of such findings for the early discrimination between pUMN and ALS groups: as observed in pLMN, the baseline progression rate of symptoms related to the motor neuron system not involved at onset—in this case, the MRC progression rate—provided the finest tool to differentiate pUMN from ALS cases with specificity values of 86%.

Moreover, the total and regional UMN burden measures exhibited excellent discriminatory performances, with sensitivity values ranging from 60 to 93% and specificity values ranging from 60 to 80%, strengthening the role of this clinical assessment tool for the early discrimination of the diverse MND phenotypes.

### 4.3. Common Observations in pLMN and pUMN Phenotypes

Both pLMN and pUMN groups did not show significant differences in terms of cognitive and behavioral features relative to ALS cases. In this context it is noteworthy to mention that, while a greater pattern of cognitive dysfunction could be expected in MND phenotypes with more prevalent cortical involvement (namely, in pUMN and ALS forms), increasing evidence points towards the existence of common cognitive/behavioral profiles in MNDs.

De Vries and colleagues have recently reported similar percentages of cognitive dysfunction in PLS, PMA, and ALS cases [22]; a finding further corroborated by the investigation of Sbrollini and colleagues, showing common language deficits in both atypical and classic MNDs [23].

Almost equal frequencies of both cognitive and behavioral changes have also been noticed and investigated across the diverse MND phenotypes [24], and specifically in pLMN patients [25], strengthening the notion that, as for ALS, pLMN and pUMN phenotypes should be reconsidered as multidomain diseases.

Intriguingly, we selectively observed in pUMN cases more severe depressive symptoms compared to classic ALS patients. This preliminary observation requires further investigation. A pilot study from Huey and colleagues has shown a relevant prevalence of depressive symptoms in PLS [26], consistent with the hypothesis that the psychosocial stress associated with MND is an important risk factor for depression, but further studies of psychiatric symptoms across the whole MND spectrum are warranted to confirm our preliminary observations.

No significant differences were observed across the diverse phenotypes in the frequency of either positive family histories for ALS and/or other neurodegenerative diseases and known genetic mutations. Again, this finding strengthens the notion of a continuum between the multiple MND classes and is further corroborated by recent studies in the field.

The mutation frequency of ALS-associated genes has been recently found to be similar in sporadic PMA and ALS cases [27], and several minor ALS-associated genes such as *ALS2*, *SETX*, *FIG4*, *OPTN*, *UBQLN2*, and *SPG11* have been consistently associated with UMN predominant phenotypes [28], suggesting a significant genetic overlap between these clinically diverse syndromes.

## 5. Conclusions

This study is not without limitations, and the first one concerns the sample size. Overall, the analyzed MND sample was indeed relatively large, but not enough to allow the finest analyses comparing individual phenotypes to be carried on. On the other hand, the nature of the study itself required the selective inclusion of patients with confirmed diagnoses over a 2-year observation period, a factor which has significantly limited the number of eligible cases.

Another shortcoming concerns the relatively reduced availability of genetic data, which was largely due to the refusal, in some patients, of the genetic screening exam.

Additionally, while the retrospective clinical charts review covered a relatively long-term interval from diagnosis, we cannot rule out the possibility of future conversion to classic disease forms in the examined pLMN and pUMN cases. In this context, while we evaluated the degree of functional impairment at the end of the study period as a proxy of the likelihood to convert to classic disease forms, additional investigations covering longer observation periods will be helpful to widen our findings.

Finally, while out of the scope of the present work, it has to be outlined that the inclusion of imaging biomarkers might be particularly useful to improve the current discrimination of MNDs, so future studies embedding such data in larger case series and with longer follow-up periods are warranted to further confirm our preliminary observations.

Despite the aforementioned limitations, this is the first study applying a multimodal characterization approach to decipher the heterogeneity of the diverse MND phenotypes and to test the added value of such a strategy for the early differentiation between more benign and more aggressive disease forms.

The obtained findings suggest that while the investigation of cognitive and genetic features across MNDs may provide new leading evidence for a neurobiological continuum, detailed clinical and neurophysiological assessments remain the elective tool to operate an early and accurate discrimination between the diverse syndromes.

These findings have the potential to facilitate patient management and prognosis estimation and to ameliorate stratification strategies for future clinical trials enrollment.

## Figures and Tables

**Table 1:** Demographic, onset, clinical, neurophysiological, extra-motor, and genetic features of classic ALS, pLMN, and pUMN patients.

	ALS (*n* = 41)	pLMN (*n* = 34)	pUMN (*n* = 19)
Demographic features			
Age (years)	65.41 (11.60)	67.44 (12.66)	63.16 (12.18)
Gender (M/F)	25/16	24/10	9/10
Education (years)	11.33 (3.45)	10.53 (4.62)	14.35 (4.27)
Onset features			
Onset side (left/right/bilateral)	21/17/3	13/14/7	6/10/3
Onset limbs (upper/lower)	23/18	17/17	4/15 *
Onset involvement (proximal/distal)	5/36	7/27	5/14
Clinical features			
Disease duration (months)	13.14 (8.95)	23.85 (30.82)	25.68 (26.94)
El Escorial Category (D/CP/ CP-LS/P/S)	9/19/12/1/0	0/2/1/1/30 **	1/4/5/9/0 **
ALSFRS-r total score (0–48)	38.04 (7.32)	36.48 (7.37)	37.00 (8.12)
ALSFRS-r rate of progression (points/month)	0.72 (0.68)	0.46 (0.43)	0.33 (0.43) *
ALSFRS-r bulbar score (0–12)	10.90 (1.85)	11.12 (1.60)	9.88 (3.01)
ALSFRS-r fine motor score (0–12)	8.07 (3.08)	7.58 (3.69)	9.35 (3.31)
ALSFRS-r gross motor score (0–12)	7.60 (3.27)	6.74 (3.08)	6.70 (2.51)
ALSFRS-r respiratory score (0–12)	11.46 (1.68)	11.03 (2.04)	11.05 (2.10)
MRC total score (0–150)	124.52 (24.28)	112.16 (24.98)	128.40 (27.82)
MRC rate of progression (points/month)	1.81 (1.69)	2.01 (2.63)	0.62 (1.27) **
MRC R UL score (0–40)	32.35 (9.17)	30.25 (10.10)	35.84 (7.10)
MRC L UL score (0–40)	32.78 (7.49)	31.25 (9.25)	33.50 (11.05)
MRC R LL score (0–35)	30.58 (6.73)	24.46 (9.11) *	30.40 (8.34)
MRC L LL score (0–35)	28.81 (8.24)	25.33 (9.18)	28.65 (9.88)
UMN total score (0–16)	7.31 (4.45)	1.37 (2.62) **	12.20 (3.29) *
UMN rate of progression (points/month)	0.65 (0.59)	0.07 (0.24) **	0.66 (0.77)
UMN UL score (0–8)	4.48 (2.77)	0.93 (2.26) **	6.93 (1.48) *
UML LL score (0–6)	2.45 (1.97)	0.41 (1.21) **	4.46 (1.40) *
UMN cranial score (0–2)	0.37 (0.68)	0.03 (0.18)	0.80 (0.77) *
Cognitive, behavioral, and mood features			
Cognitive phenotype (motor/MND-CBI)	24/11	21/8	10/4
Total ECAS score (0–136)	105.73 (16.56)	99.93 (19.76)	110.84 (9.09)
ALS specific functions (0–100)	79.61 (13.42)	74.56 (16.19)	83.30 (7.59)
Executive functions (0–48)	36.76 (6.95)	33.25 (9.83)	37.23 (5.47)
Language functions (0–28)	24.34 (3.70)	22.87 (4.24)	26.07 (1.75)
Verbal fluency (0–24)	18.40 (5.12)	18.43 (4.33)	20.00 (2.82)
ALS non-specific functions (0–36)	26.20 (4.62)	25.37 (5.73)	27.53 (2.96)
Memory functions (0–24)	14.54 (4.38)	14.09 (5.25)	15.84 (2.99)
Visuospatial functions (0–12)	11.65 (0.72)	11.28 (1.19)	11.69 (0.63)
ECAS carer behavior screen (0–10)	0.38 (0.65)	0.37 (0.56)	0.58 (1.16)
HADS total score (0–42)	6.51 (5.66)	5.85 (4.06)	11.25 (5.15) *
HADS depression score (0–21)	2.82 (2.95)	2.81 (2.11)	5.58 (3.08) *
HADS anxiety score (0–21)	3.68 (3.30)	3.03 (2.48)	5.66 (3.02)
Familiarity and genetic features			
Familiarity (no/yes)	31/10	26/8	10/9
Genetic mutations (no/yes)	13/6	15/5	7/1
Neurophysiological features			
CMAP amplitude R median nerve (μV)	2.25 (2.36)	2.25 (2.05)	NA
CMAP amplitude L median nerve (μV)	1.44 (1.78)	3.10 (4.10)	NA
CMAP amplitude R ulnar nerve (μV)	5.07 (1.85)	6.25 (3.21)	8.10 (1.95) *
CMAP amplitude L ulnar nerve (μV)	4.99 (2.82)	5.31 (3.19)	5.76 (1.68)
CMAP amplitude R peroneal nerve (μV)	3.26 (2.23)	2.09 (2.12)	3.61 (1.59)
CMAP amplitude L peroneal nerve (μV)	2.56 (2.27)	1.85 (1.67)	4.22 (1.62)
CMAP amplitude R tibial nerve (μV)	4.00 (4.60)	1.80 (0.96)	6.95 (3.60)
CMAP amplitude L tibial nerve (μV)	1.93 (1.45)	4.01 (4.93)	8.90 (3.95)
MEP CMCT R abductor pollicis brevis (ms)	9.39 (1.41)	8.35 (2.63)	10.27 (1.88)
MEP CMCT L abductor pollicis brevis (ms)	7.64 (1.52)	7.48 (1.53)	7.99 (1.40)
MEP CMCT R tibialis anterior (ms)	15.23 (5.65)	15.19 (2.25)	30.66 (22.88) *
MEP CMCT L tibialis anterior (ms)	16.54 (6.65)	13.84 (2.16)	17.99 (4.43)

** *p* < 0.001, * *p* ≤ 0.05 relative to classic ALS. Abbreviations: ALS = classic amyotrophic lateral sclerosis; ALSFRS-r = ALS Functional Rating Scale-revised; CMCT = central motor conduction time; CMAP = compound muscle action potential; CP = clinically probable; CP-LS = clinically probable–laboratory supported; D = definite; ECAS = Edinburgh Cognitive and Behavioral ALS screen; F = females; HADS = Hospital Anxiety and Depression Scale; L = left; LL = lower limbs; M = males; MEP = motor-evoked potential; MRC = Medical Research Council; P = possible; pLMN = pure/predominant lower motor neuron; pUMN = pure/predominant upper motor neuron; R = right; S = suspected; UL = upper limbs.

**Table 2:** Sensitivity and specificity in differentiating between ALS and pLMN patients.

Measure	AUC (%)	AUC Category	Cut-Off	Sensitivity (%)	Specificity (%)
MRC R LL	67	ND	28.00	61	84
UMN total score	89	E	0.50	72	97
UMN rate of progression	90 *	E	0.05	83	89
UMN UL	84	E	0.50	83	86
UMN LL	81	E	1.50	90	69

* The finest tool to discriminate between ALS and pLMN. Abbreviations. A = acceptable; AUC = area under curve; E = excellent; LL = lower limbs; MRC = Medical Research Council; ND = no discrimination; R = right; UL = upper limbs; UMN = upper motor neuron.

**Table 3:** Sensitivity and specificity in differentiating between ALS and pUMN patients.

Measure	AUC (%)	AUC Category	Cut-Off	Sensitivity (%)	Specificity (%)
ALSFRS-r rate of progression	74	A	0.31	82	68
MRC rate of progression	82 *	E	0.38	69	86
UMN total score	81	E	10.50	73	77
UMN UL	77	A	7.50	60	80
UMN LL	78	A	2.50	93	60
UMN cranial	66	ND	0.50	60	74
HADS depression score	80	E	2.50	100	59
HADS total score	76	A	7.50	83	66
CMAP amplitude R ulnar nerve	79	A	8.95	60	94
MEP CMCT R tibialis anterior	75	A	17.46	88	64

* The finest tool to discriminate between ALS and pUMN. Abbreviations. A = acceptable; ALSFRS-r = ALS Functional Rating Scale-revised; AUC = area under curve; CMCT = central motor conduction time; CMAP = compound muscle action potential; E = excellent; HADS = Hospital Anxiety and Depression Scale; LL = lower limbs; MEP = motor-evoked potential; MRC = Medical Research Council; ND = no discrimination; R = right; UL = upper limbs; UMN = upper motor neuron.

**Table 4:** Regression analyses linking baseline differentiating measures to ALSFRS-r scores at the end of the study period.

**pLMN Patients**				
ALSFRS-r at 2 Years	Tested Predictor	Estimate	T Value	*p* Value
31.38 ± 9.15	MRC R LL	0.38	1.94	0.05 *
	UMN total score	−0.05	−0.05	0.95
	UMN rate of progression	−3.78	−0.06	0.95
	UMN UL	−0.53	−0.43	0.66
	UMN LL	−0.50	−0.33	0.74
**pUMN Patients**				
ALSFRS-r at 2 Years	Tested predictor	Estimate	T Value	*p* Value
29.91 ± 6.80	ALSFRS-r rate of progression	−9.10	−2.82	0.01 *
	MRC rate of progression	−2.95	−2.78	0.02 *
	UMN total score	0.53	0.72	0.49
	UMN UL	1.16	0.67	0.51
	UMN LL	1.05	0.64	0.53
	UMN cranial	2.50	0.68	0.51
	HADS depression score	1.13	1.58	0.15
	HADS total score	0.25	0.52	0.61
	CMAP amplitude R ulnar nerve	1.63	0.55	0.68
	MEP CMCT R tibialis anterior	−0.03	−7.55	0.08

* *p* ≤ 0.05. Abbreviations: ALSFRS = ALS Functional Rating Scale-revised; CMCT = central motor conduction time; CMAP = compound muscle action potential; HADS = Hospital Anxiety and Depression Scale; L = left; LL = lower limbs; MEP = motor-evoked potential; MRC = Medical Research Council; pLMN = pure/predominant lower motor neuron; pUMN = pure/predominant upper motor neuron; R = right; UL = upper limbs.

## Data Availability

Raw data are available upon appropriate request.

## Supplementary Materials

The following supporting information can be downloaded at: https://www.mdpi.com/article/10.3390/life12101506/s1, Table S1. List of genes included in the panels.
