# Supplementary material for: MND Phenotypes Differentiation: The Role of Multimodal Characterization at the Time of Diagnosis
Source: Life (Basel). 2022 Sep 27;12(10):1506. doi: 10.3390/life12101506 (PMC9604895; doi:10.3390/life12101506)
Supplement: Supplementary file 1 [file life-12-01506-s001.zip › life-1900886-supplementary.pdf]

Supplementary Table S1. List of genes included in the panels.

Panel 1 genes		Panel 2 genes	
ALS2	OPTN	ANXA11	NEK1
ANG	PFN1	C19orf12	PLD3
BSCL2	PSEN1	CCNF	PRPH
CHMP2B	PSEN2	CHCHD10	SIGMAR1
DCTN1	SETX	CTSF	SQSTM1
ERBB4	SOD1	CYP27A1	SS18L1
FIG4	SPG11	DAO	TAF15
FUS	TARDBP	DJ1	TBK1
GRN	UBQLN2	ELP3	TIA1
HNRNPA1	VAPB	EPB41L1	TREM2
MAPT	VCP	EWSR1	TUBA4A
MATR3		GLE1	UNC13A
		HNRPA2B1	VRK1
		LMNB1	
